# Electrographic Waveform Structure Predicts Laminar Focus Location in a Model of Temporal Lobe Seizures *In Vitro*

**DOI:** 10.1371/journal.pone.0121676

**Published:** 2015-03-23

**Authors:** Christopher Adams, Natalie E. Adams, Roger D. Traub, Miles A. Whittington

**Affiliations:** 1 Institute of Neuroscience, Newcastle University, Newcastle upon Tyne, United Kingdom; 2 Hull York Medical School, The University of York, York, United Kingdom; 3 Dept. Physical Sciences, IBM TJ Watson Research Center, New York, New York, United States of America; 4 Department of Neurology, Columbia University, New York, New York, United States of America; Consejo Superior de Investigaciones Cientificas—Instituto Cajal, SPAIN

## Abstract

Temporal lobe epilepsy is the most common form of partial-onset epilepsy and accounts for the majority of adult epilepsy cases in most countries. A critical role for the hippocampus (and to some extent amygdala) in the pathology of these epilepsies is clear, with selective removal of these regions almost as effective as temporal lobectomy in reducing subsequent seizure risk. However, there is debate about whether hippocampus is ‘victim’ or ‘perpetrator’: The structure is ideally placed to ‘broadcast’ epileptiform activity to a great many other brain regions, but removal often leaves epileptiform events still occurring in cortex, particularly in adjacent areas, and recruitment of the hippocampus into seizure-like activity has been shown to be difficult in clinically-relevant models. Using a very simple model of acute epileptiform activity with known, single primary pathology (GABA_A_ Receptor partial blockade), we track the onset and propagation of epileptiform events in hippocampus, parahippocampal areas and neocortex. In this model the hippocampus acts as a potential seizure focus for the majority of observed events. Events with hippocampal focus were far more readily propagated throughout parahippocampal areas and into neocortex than vice versa. The electrographic signature of events of hippocampal origin was significantly different to those of primary neocortical origin – a consequence of differential laminar activation. These data confirm the critical role of the hippocampus in epileptiform activity generation in the temporal lobe and suggest the morphology of non-invasive electrical recording of neocortical interictal events may be useful in confirming this role.

## Introduction

A role for the hippocampus in temporal lobe epilepsies is clear. Local circuit properties—particularly recurrent excitatory synaptic connection density in area CA3 [1] and critical dependence on strong GABA-ergic inhibition in areas CA2/CA1 [2] allow local circuits to generate intense, hypersynchronous discharges under the influence of epileptogenic pathology [3]. Once initiated such discharges spread through area CA1 and can then be widely ‘broadcast’ to a multitude of cortical and sub-cortical regions [4]. It is not surprising then that, in patients with unitemporal seizure origin almost 90% are seizure-free following surgical hippocampal removal [5]—a rate as good, if not better than temporal lobectomy but far less iatrogenic damage. However, this remaining ca. 10% of patients in whom epileptiform activity persists after hippocampectomy fuel the debate about whether the hippocampal formation is victim or perpetrator in epilepsy. Hippocampal recruitment *de novo* following sclerosis is difficult, with very severe damage predicted to be required before the hippocampus itself acts as a source (rather than just a distributor) of epileptiform activity [6]. Propagation paths for aberrant activity out of hippocampus are not clear—following status epilepticus further seizure-like events have multiple apparent focal origins with variable and discontiguous patterns of propagation from them [7].

Further complicating this picture is the suggestion that hippocampus proper and parahippocampal areas both have equal epileptogenic potential [7]. In addition, models of epileptiform activity in these brain regions reveal different patterns of seizure focus and spread apparently depending on the nature of the pathology introduced [8,9]. For example, the low magnesium model of epileptiform activity involves both boosting NMDA receptor-mediated excitatory neurotransmission and also reducing GABA_A_-receptor mediated inhibition [10]. Similarly the 4-AP model involves blockade of neuronal repolarisation via Kv1 potassium channels [11], but also—at least in hippocampus—the formation of depolarising, excitatory GABAergic network activity [12]. This suggests a need to quantify seizure origin and spread within these areas, using models that introduce specific and well-documented primary pathology are needed.

Here we use a very basic acute seizure model (selective GABA_A_ receptor-mediated disinhibition *alone*) combined with voltage sensitive dye imaging to investigate further the origin of epileptiform activity and its spread along the hippocampal-neocortical axis.

## Methods

Data were obtained from horizontal slices, 0.45 mm thick, from normal (non-epileptic) adult male Wistar rats obtained from B&K Universal, housed in pairs and given free access to food and water prior to terminal anaesthesia (Isoflurane followed by ketamine/xylazine). Slice preparation was briefly as follows: Terminally anaesthetised rats were intracardially perfused with buffered, ice-cold sucrose solution. The brain was removed and transferred to a vibratome. Resulting slices were maintained at 34°C at the interface between warm, wet 95% O_2_/ 5%CO_2_ and a perfusate of artificial cerebrospinal fluid (aCSF) containing (in mM): 126 NaCl, 3 KCl, 1.25 NaH_2_PO_4_, 1 MgSO_4_, 1.2 CaCl_2_, 24 NaHCO_3_ and 10 glucose. Spontaneous epileptiform events were induced by bath application of bicuculline (0.1–0.2 mM). All tissue preparation was performed in accordance with the UK Animals (Scientific Procedures) act 1986 and with consent from the University of York Animal Ethics Committee.

Electrographic data were recorded as local field potentials using glass micropipettes filled with aCSF (resistance 0.1–0.5 MΩ), digitised at 2 kHz and bandpass filtered at 0.5–100 Hz). Spatiotemporal patterns of activity were studied by loading slices with the voltage-sensitive dye (VSD) di-8-ANEPPS in 0.5% ethanol/DMSO containing aCSF fluid for 1–3h. Slices were then illuminated through a 10x objective by 532 nm (Coherent) laser and changes in output spectra recorded in the red range at 200 Hz using a Micam Ultima CCD camera (100 x 100 pixels, 25 mm square). Only a maximum of 6 x 10s epochs of data were taken from each slice to prevent result bias from bleaching under laser light. Fluorescence data were converted to 16 bit, grayscale TIFF stacks and exported to Matlab for analysis: Spatially, each frame was background-subtracted, detrended and ‘post’-filtered at 2x2 pixels. Each pixel was then temporally manipulated using a Savitzky-Golay differentiation filter. Seizure wavefronts were detected as positive crossings of a threshold set at 2SD above baseline (non-epileptiform) activity. Velocity of propagation measurement were estimated (no compensation for curvature of structures was used) from the spread of these wavefronts across the slices. Wavefront data was rendered in 3D-space (x-, y- slice coordinates and time) using the Iso2mesh toolbox [13]. All raw data and bespoke analysis routines are available on the ‘CARMEN’ website (Carmen.org.uk).

## Results

**Fig 1:**
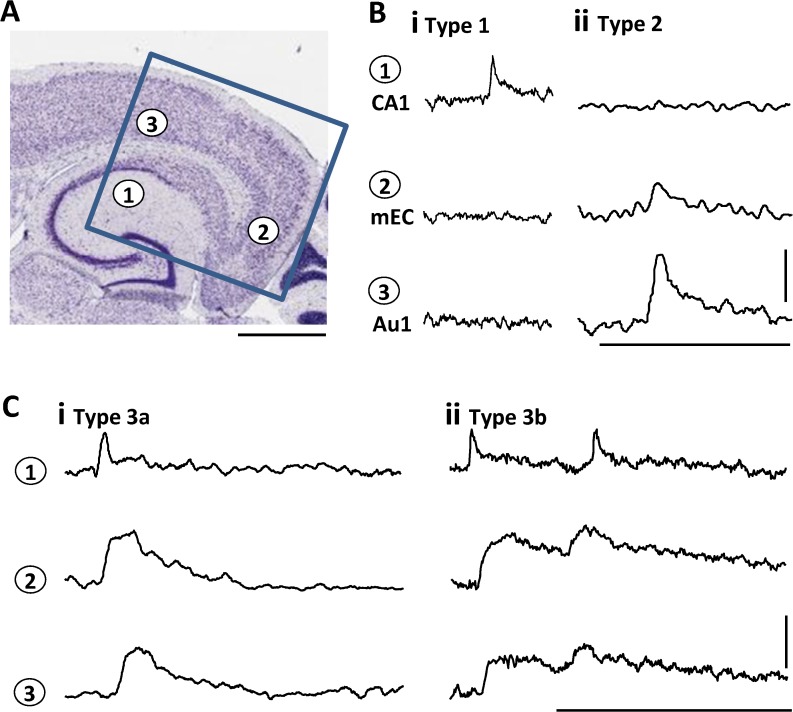
Example VSD recordings of transient, spontaneous epileptiform discharges at three sites along the hippocampal—neocortical axis. **A.** Cartoon illustrating the 3 sites the example fluorescence changes illustrated were taken from: 1—stratum radiatum of area CA1 of the hippocampus, 2—layer 5/6 of medial entorhinal cortex (mEC), 3—layer 5/6 of auditory cortex (Au1). Box shows the coverage of the CCD chip used to record the data. Scale bar 1 mm. **B/C**. Examples of the 4 subtypes of spatiotemporal, interictal activity seen, each example is 2 x 2 pixel binned and temporally filtered (see methods). **Bi.** Non-propagating events originating in hippocampus. **Bii**. Non-propagating events originating in Au1 (note amplitude-degraded event visible in mEC but not hippocampus). **Ci**. Propagating, reverberating events originating in hippocampus, projecting to neocortex and returning to hippocampus. **Cii**. Propagating, non-reverberating events originating inhippocampus. Scale bars B/C 0.1% Δf, 2 sec.

57 spontaneous interictal events lasting 0.2–2.8s were captured from 25 slices from 12 rats. Events were not stereotyped in terms of amplitude or origin and were divided here into 4 main types depending on region of initiation and pattern of propagation (Fig. 1). These were as follows: Type 1, Non-propagating events originating in hippocampus (14/57). Type 2, Non-propagating events originating in primary auditory cortex (Au1, 11/57). Type 3a, propagating events originating in hippocampus, projecting through subiculum, medial and lateral entorhinal cortices and perirhinal cortex to neocortex (30/57). Type 3b, a subset of type 3a events originating in hippocampus, also projecting to neocortex but then returning to hippocampus through each intermediate structure along the hippocampal-neocortical axis (7/30). In only two cases were events captured that originated in intermediate structures—both arising in deep layers of medial entorhinal cortex. In no cases were events captured that originated in neocortex and projected to hippocampus. In order to understand the signatures of each event that corresponded to each type of dynamic behaviour we first considered differences within hippocampus.

### Hippocampal spatiotemporal profiles of propagating and non-propagating interictal-like events

**Fig 2:**
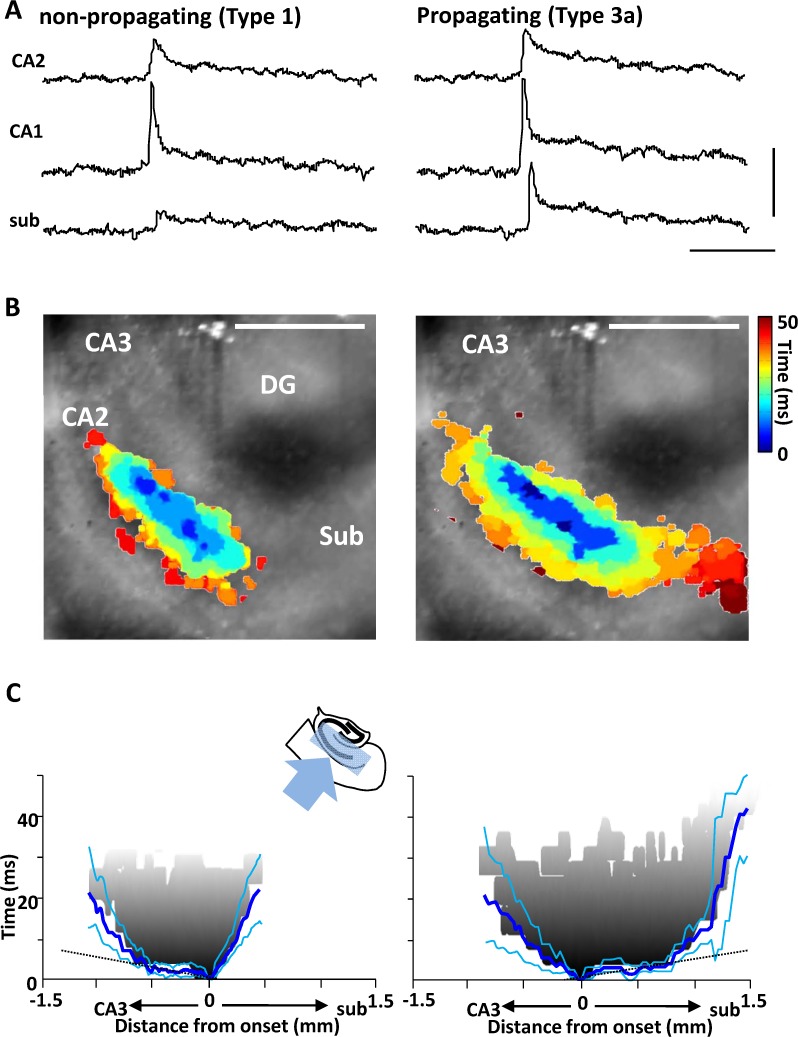
Spatiotemporal properties of non-propagating vs. propagating events originating in hippocampus. **A.** Example interictal fluorescence changes along the CA axis for non-propagating and propagating events. Not both types of event originate in CA1 (towards the CA2 border), both types propagate retrogradely into CA3, but only propagating events successfully invade subiculum. Scale bar 0.1% Δf, 1 sec. **B**. Spatiotemporal maps of the two event types in hippocampus. Only areas demonstrating activity over threshold are represented on the colormap, overlaid on the transmitted light (non-fluorescent) slice image. Main areas are labelled DG (dentate gyrus), CA2,3 (cornu ammonis subdivisdion 2,3), Sub (subiculum). Note the activity is concentrated in mid stratum radiatum. Colormap represents time from event onset (cool-hot). **C**. Mean event onset and spread (n = 6 slices) viewed along the CA1 horizontal axis (as illustrated by the cartoon insert). An individual example of an interictal wavefront (1^st^ threshold crossing) is shown in gray, blue lines show mean ± s.e.mean. Note the mean initial propagation velocity is very rapid along CA1 (slope of wavefront, dotted lines) in opposite directions for the two events, but in each case, velocity slows markedly on reaching CA3 and subiculum (see results for quantification).

Type 1 and type3a&b events both always began at the CA1/CA2 border before rapidly spreading in both directions along the cornu ammonis to area CA3 and along the CA1 subregion (Fig. 2). The majority (72%) of captured interictal-like events began like this. Each subtype exhibited maximum percentage fluorescence change at the origin (0.14 ± 0.22 (type 1), 0.16 ± 0.15 (Type 3a), 0.12 ± 0.24 (Type 3b), P>0.1, n = 14, 23 & 7 respectively, Fig. 2A). In addition, no significant difference was found in the extent and speed of spread through CA3. Mid-CA3 percentage fluorescence changes were degraded slightly from those at the focus (0.10 ± 0.03 (type 1), 0.08 ± 0.02 (Type 3a), 0.08 ± 0.03 (Type 3b), P>0.1, n = 14, 23 & 7 respectively, Fig. 2A). Mean rate of spread of the wavefront was between 0.03 and 0.05 m.s^-1^ at 34°C in each case (Fig. 2C). However, quantifiable differences in the pattern of spread along area CA1 were observed when comparing events that remained in hippocampus (type 1) and those that propagated to neocortex (types 3a/b). Non-propagating type 1 events projected slowly (0.17 ± 0.03 m.s^-1^) for only ca. 0.5 mm along CA1 whereas type 3a/b events propagated along the entire CA1 axis to subiculum at an initial, rapid rate (0.37 ± 0.07 m.s^-1^, P<0.05 cf non propagating events along CA1) before slowing at the CA1-subiculum border (Fig. 2C).

Type 3 events fell into two subcategories: those that projected to neocortex and terminated, and those that projected to neocortex and then back to area CA1. We therefore next compare the spatiotemporal dynamics of these forward (hippocampus—neocortex) and back (neocortex to hippocampus) propagations.

### Pattern of propagation through periallocortex

**Fig 3:**
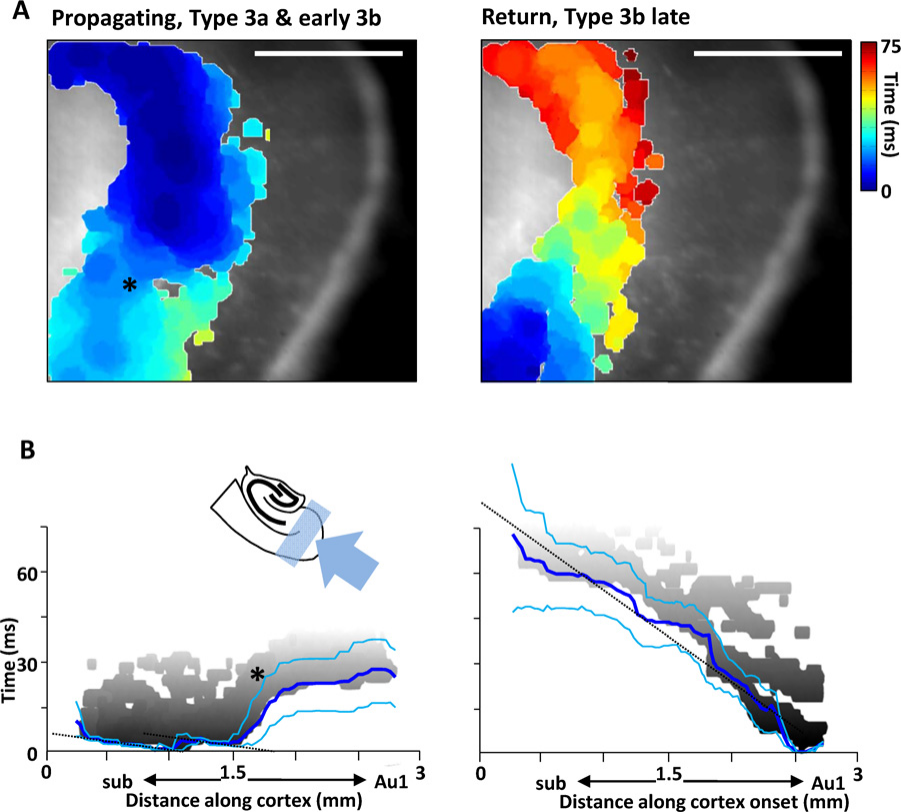
Comparison of propagation dynamics for events of hippocampal origin propagating to neocortex, and the reverberative return wave. **A**. Spatiotemporal maps of activity spread through subiculum (top left of the field of view), medial & lateral entorhinal cortex and perirhinal cortex towards Au1. Only areas demonstrating activity over threshold are represented on the colormap, overlaid on the transmitted light (non-fluorescent) slice image. Note the activity is concentrated in deep cortical layers only. Colormap represents time from event onset (cool-hot). Scale bar 1mm. **B**. Mean event spread (n = 6 slices) viewed along the periallocortical horizontal axis (as illustrated by the cartoon insert). ‘Zero’ distance is set for the CA1/subiculum border. Note this is different from Fig. 2C as the majority of area CA1 was not present in the view field for these experiments. An individual example of an interictal wavefront (1^st^ threshold crossing) is shown in gray, blue lines show mean ± s.e.mean. Note the rapid, saltatory-like spread of activity from hippocampus through periallocortex (the ‘jump’ in activity position with time followed by both forward and back (shown as dotted lines) propagation instead of just monotonic forward propagation) and the very slow conduction from lateral entorhinal to perirhinal cortices (asterisk in **A** and **B**). In contrast, the reverberative return wave from Au1 to hippocampus was relatively monotonic.

Propagation of interictal-like events from hippocampus through periallocortex was rapid and exhibited saltatory properties—discrete ‘jumps’ of activity from one locus in the general propagation direction to a more distal locus, followed by both forward and back propagation (Fig. 3, left panel). It involved recruitment of deep layers exclusively. From hippocampus activity jumped to the distal end of medial entorhinal cortex (mEC) before jumping further to the distal end of lateral entorhinal cortex (lEC). After each jump, activity propagated rapidly back towards subiculum (0.47 ± 0.11 ms^-1^, Fig. 3B). From lEC an apparent boundary to propagation was suggested by the considerable slowing of further propagation through peririnal cortex (0.02 ± 0.11 m.s^-1^, see discussion). Overall propagation time from CA1 focus to deep layers of primary auditory neocortex (Au1) was 25 ± 8 ms (n = 30) for all type 3 events.

In complete contrast, interictal-like events propagating back to hippocampus from previously invaded neocortex showed near monotonic, considerably slower conduction speeds and no evidence for saltatory conduction despite also exclusively utilising connectivity within deep cortical layers (Fig. 3A, B). Overall return conduction time from Au1 to area CA1 was 74 ± 15 ms (P<0.05 cf propagation times from hippocampal events described above, n = 7). These reverberatory events were initiated in Au1 on the decay of the fluorescence change (membrane potential depolarisation) caused by the initial invasion of neocortex by activity originating from area CA1 (Fig. 1C). The 7 events captured began 0.42–0.78 seconds after the initial invasion, at a time when the fluorescence signal had still not decreased to half its initial maximum (see discussion).

### Laminar structure of events of neocortical or hippocampal origin

**Fig 4:**
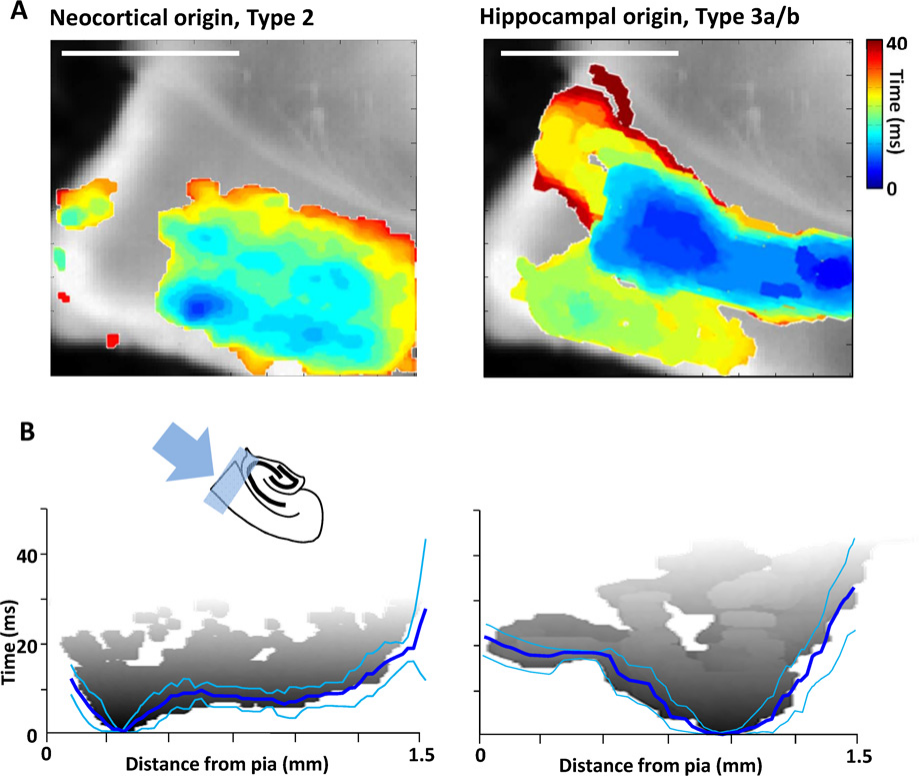
Comparison of propagation dynamics within neocortex. **A.** Spatiotemporal maps of activity spread through neocortical laminae for events originating in cortex (Type 2 events, left panel) and those spreading to cortex from hippocampus (Type 3 events with (3b) or without (3a) backpropagation, right panel). Only areas demonstrating activity over threshold are represented on the colormap, overlaid on the transmitted light (non-fluorescent) slice image. Colormap represents time from event onset (cool-hot). B. Mean event spread (n = 6 slices, left panel, n-5 slices right panel) viewed along the radial cortical axis from pia to subcortical while matter (as illustrated by the cartoon insert). An individual example of an interictal wavefront (1^st^ threshold crossing) is shown in gray, blue lines show mean ± s.e.mean. Note the superficial focus for events of neocortical origin and the deep focus for events propagating from hippocampus.

In addition to the majority events with a hippocampal focus a number of interictal-like events were seen to originate in Au1. Of the 11 type 2 events captured none of them resulted in activity propagating to hippocampus. This was in contrast to the reverberatory events described above, arising from the initial activation of neocortex by hippocampus. In an attempt to understand why events with Au1 foci did not propagate outside neocortex we studied in more detail their local dynamics. Initial observation revealed that the two types of cortical activity were initiated in completely different layers within Au1 (Fig. 4A): 11/11 events with Au1 focus initiated in layer 2/3 whereas all 30 events projected from hippocampus began in layers 5/6. Interestingly, of these 30 events, only the 7 that resulted in reverberatory activity back to hippocampus also generated fluorescence changes above threshold in superficial layers.

Interictal-like events with origin in Au1 rapidly projected from layer 2/3 to layer 5 with a conduction time of 8 ± 2 ms (n = 11, Fig. 4B). Projection to deeper layers became progressively slower and unreliable. The events remained horizontally confined to a spread of 1.5 ± 0.4 mm (layers 2/3) and 1.6 ± 0.6 mm (layers 5/6) (n = 11, P>0.1) around the focus with a distinct bias to propagation towards periallocortex rather than more rostral associational and somatosensory areas (see Fig. 4A, left panel). The 7 events captured that originated in hippocampus *and* recruited superficial Au1 had opposite laminar profiles to those described above. Origin was always in layers 5/6 with a slower conduction time to layers 2/3 of 17 ± 3 ms (n = 7, P<0.05 cf conduction in the opposite direction for events of Au1 origin). Two additional features were of note: Firstly, once activated, horizontal propagation within layers 2/3 was near-identical to that seen for events of Au1 origin. Spread was 1.3 ± 0.3 mm with, again, a distinct bias to propagation towards periallocortex. Secondly, activity in deep layers was ‘en passant’—propagating to Au1 through the deep layers and continuing out of Au1 during and after activation of layers 2/3 (Fig. 4B, right panel). These contrasting local dynamic signatures of interictal-like events—particularly their laminar differences—suggested that they should be distinguishable with conventional electrophysiological measures of local field potentials.

### Consequences for electrographic recordings

**Fig 5:**
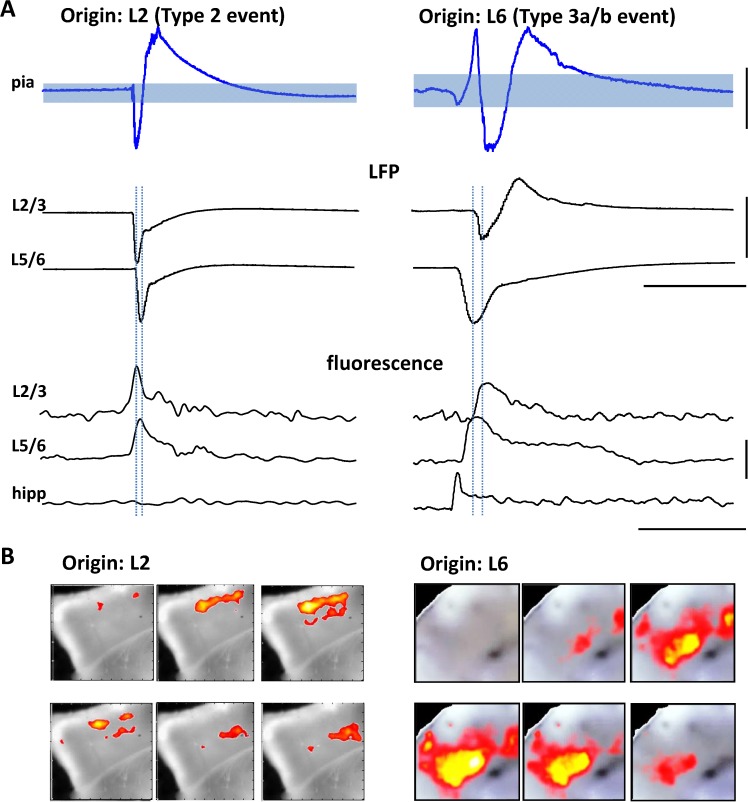
Laminar origin of local and hippocampally-projected neocortical discharges is reflected in the electrographic waveform structure. **A. Upper traces.** Average of 12 events measured with an extracellular electrode at the pia to model non-invasive cortical recordings (pia). Shaded region represents +/- 2 SD of the mean trace shown. Note the overt biphasic vs. triphasic shape of the electrographic events when comparing interictal-like discharges of neocortical vs. hippocampal origin respectively. **Middle traces.** Concurrent superficial (L2/3) and deep (L5/6) cortical local field potential (LFP) recordings showing different laminar onsets. **Lower traces.** Example 2x2-binned fluorescence changes for the two event types. Scale bars 20μV (upper), 0.2 mV (middle), 0.1% ΔF (lower), 0.5 sec. **B**. Selected frames from the events in A showing the sequence of spatiotemporal activation for the two interictal-like events. Note the more overt, diffuse activation of deep layers for events propagated from hippocampus.

Comparison of fluorescence changes concurrently with local field potential (LFP) recordings from the pial surface of Au1 were used to relate the spatiotemporal pattern of interictal-like events to a surrogate for intra- or extra-cranial recordings *in vivo* (Fig. 5). When comparing type 2 events of primary neocortical origin with type 3b events of hippocampal origin we found a distinct difference in the shape of the superficial LFP: Type 2 events that originated in superficial layers of Au1 were accompanied by biphasic LFPs with initial negative-going deflections. With a threshold set at +2 standard deviations (SD) from the trace mean there were always 2 threshold crossings for this event type. Mean line length from first to last crossing (filtered data) was 1.17 ± 0.12 s (n = 14 events). Mean peak-peak amplitude deflection of the pial signal was 42 ± 10 μV (n = 5, Fig. 5A, upper panel). Paired LFP recordings from layers 2/3 and 5/6 during these events of auditory origin confirmed the propagation from superficial to deep neocortex as seen with the fluorescence recordings (Fig. 5A, mid and lower panels).

In contrast type 3a/b events, with hippocampal origin, were associated with pial LFPs showing a triphasic form and initial positive-going deflection. There were always 4 threshold crossings when threshold was set to +2SD of the traces. Mean line length from first to last threshold crossing was 1.62 ± 0.19 s (n = 30, P<0.05 cf. line lengths for type 2 events in auditory cortex above). Mean peak-peak amplitudes were not significantly different from the biphasic events with primary Au1 origin (46 ± 12 μV, n = 5, P>0.1). Again, paired LFP recordings from layers 2/3 and 5/6 confirmed the deep to superficial propagation of this type of event within neocortex (cf. Fig. 4B). In addition to the different laminar origin of the type 2 and type 3a/b auditory cortical events the type 3 events were associated with a longer duration of fluorescence change (depolarisation) in layers 5/6; Type 2, 0.18 ± 0.09 s vs. type 3a/b, 0.70 ± 0.15 s (P<0.05).

## Discussion

These data confirm the dominant role for hippocampus in most temporal lobe epilepsies. While the model used here is simplistic at best it clearly showed that, within the hippocampal-neocortical axis, most epileptiform events (over 70%) had a focus along the CA3/CA1 boundary [2]. As with clinical observations following hippocampectomy, a small number of events of primary neocortical origin were also present, however, these were seen to be highly localised and of extremely low risk of spread and recruitment of other brain regions. In contrast, two thirds of the events demonstrating a hippocampal focus effectively recruited all the periallocortical areas present in the slice as well as proximal neocortical regions. In addition, there was a risk of repetitive activation of hippocampus via reverberation of these projected events. Pairing superficial layer local field potential traces with the VSD data showed than events of neocortical origin had biphasic voltage waveforms whereas events of hippocampal origin had triphasic (spike and wave-like) waveforms (Fig. 5). Given the difficulty in directly recording hippocampal events with conventional, non-invasive EEG methods [14], these data suggest the form of interictal cortical surface electrographic activity may be of use in determining causal nature of hippocampal pathology in temporal lobe epilepsies.

The origin and spread within hippocampus seen here is largely consistent with previous reports on epileptiform events in acutely disinhinited hippocampus [2,15]. However, different tissue preparations have been shown to yield different foci within hippocampus. Transverse *in vitro* hippocampal slice work (slices in this study were horizontal) has shown that epileptiform events can occur at either end of area CA3 [16, 17] with GABAergic disinhibition alone favouring area CA2/CA3a [18]. In each case area CA1 activation had an absolute requirement of CA3 involvement. As these and the present study all used disinhibition models this may reflect the starkly different patterns of network inhibition seen transversely compared to horizontally in the hippocampus proper [19]. For example, a distinct role for area CA2 in shaping propagatory events from DG/CA3 has been demonstrated with inhibition intact [20]. This was difficult to explore further here as we used a disinhibition model and also did not routinely record from area CA3c and the hilus. With intact hippocampal circuitry disinhibition-induced epileptiform activity has been shown to originate further along area CA1 towards subiculum, further complicating the issue of precise origin of epileptiform activity within the hippocampal circuit [21].

The incidence of both hippocampal origin and probability of event propagation out of hippocampus were highly consistent with seizures seen in medically refractory temporal lobe epilepsies. In the clinical case approximately 80% of seizures had a hippocampal focus and 60–80% of these spread to neocortex [22,23]. The reasons for this lack of stereotypy in event propagation may stem from the nature of the primary pathology underlying the seizures and the way this is modelled. In particular, potassium conductances appear to play a role. In the present study only GABA_A_ receptors were partially blocked, leaving GABA_B_ receptors intact. While this form of inhibition has not been shown to affect seizure induction and spread in neocortex [24], it has been shown to be involved in termination of seizures within the hippocampal formation [25].

Differences in propagation trajectories seen in the present study compared with similar *in vitro* studies also suggest the role for potassium conductances involved in afterhyperpolarisations in principal cells and their resting membrane potentials. In the present study only deep layers of periallocortical regions were recruited and acted as channels for hippocampal event spread (and return, Fig. 3). Models using disinhibition combined with lower magnesium ion concentration (to boost NMDA receptor-dependent synaptic excitation), or 4-aminopyridine to block Kv1-subtype of potassium channels [11] have shown recruitment of more superficial entorhinal cortex and dentate gyrus [9,10]. In addition, region-specific differences in the balance of synaptic inhibition and excitation has also been suggested to account for both propagation trajectory patterns and dynamics and whether focal seizures spread at all [26]. Changes in microcircuit connectivity may also account for the saltatory nature of propagation of hippocampal events to neocortex as previously suggested [27], but do not appear to provide insight into the directionality of this phenomenon seen here: Saltatory propagation trajectories were only seen in the hippocampal-to-neocortical direction and not vice versa (Fig. 3).

Of particular importance for reverberative activity is the dentate gyrus. This region is activated by layer 2 entorhinal cortical neurons via the perforant path, rather than the direct temporoammonic pathway to area CA1 originating in layer 3 and deep layers as seen here [28]. The dentate gyrus is remarkably resistant to seizure spread, not because of GABA_A_ receptor-mediated inhibition (as reduced in this study), but because of a highly hyperpolarised, potassium channel-dependent resting membrane potential [29]. With acute disinhibition alone, as used here, the seizure-resisting effects of intrinsic potassium conductances in superficial periallocortical and dentate gyral neurons would be intact. In the pilocarpine model of epilepsy this is manifest as a preservation of the filtering properties of the dentate gyrus for neocortical input to hippocampus via the performat path [30]. Interestingly this study also showed a huge (ca. 10-fold) increase in excitatory effects of temporoammonic inputs directly to area CA1—the only active trajectory seen in the present study for re-entrant activity into hippocampus. However, in models of chronic hyperexcitability the bombardment of dentate gyrus with epileptiform events of cortical origin gradually induces cell death. Only when this becomes extreme does the hippocampus act as a channel for repetitive epileptiform activity [6]. However, this may not invalidate the model used in the present study. In approximately 30% of cases of drug intractable temporal lobe epilepsy no lesions were seen but hippocampectomy was to a large extent successful [31], suggesting sclerosis is not a prerequisite for a key role for hippocampus in seizure generation.

The difference in propagation trajectories for hippocampofugal compared to hippocampopetal events suggested different constraints for seizure spread to and from this region. The very rapid, saltatory spread from CA1 through entorhinal cortices may reflect the recruitment of physiologically relevant pathways for hippocampal communication with neocortex. However, the apparent, at least partial, barrier formed by the perirhinal cortex suggests a critical role in seizure spread from hippocampus. In general perirhinal cortex is spared in mesial temporal lobe epilepsy. However, if epileptiform activity is present here it can be severe [32]. It has been shown to be a highly sensitive area for intervention for seizure propagation and resistance to kindling [33,34] but is rarely removed in epilepsy surgery [35].

Within neocortex the fate of interictal epileptiform events was seen to be very different depending on whether the events were of neocortical origin or projected from hippocampus. In the former case (type 2 events here) events always started in superficial layers as seen for a number of acute epileptiform activity models (eg. Ref [36], but see also ref [37]). Projection to deep layers was rapid and seen for all events. In contrast, type 3 events invading neocortex from a hippocampal focus began in deep layers, as has been shown to occur occasionally in zero magnesium models [37]. Projection to superficial layers was seen in less than 50% of cases and, when present, occurred relatively slowly. This may reflect the ca. 5-fold less excitatory connectivity from deep to superficial cf. superficial to deep layers seen in primary sensory neocortex [38]. This imbalance between descending and ascending interlaminar connections may also, in part, explain the occurrence of reverberative epileptiform events: These were only seen in cases where superficial layers were activated. The slow conduction times to superficial layers may ensure that subsequent reactivation of deep layers occurs at an ideal time while neurons in layers 5/6 are no longer refractory, but are still relatively depolarised. In addition, this re-entrant activation of deep layers appeared also to underlie the different electrographic shapes of neocortically recorded events of local or hippocampal origin (Fig. 5). This dependence on seizure origin for seizure discharge shape has been noted in other models [39], where deep (ventriculocisternal) origins had more ‘spike and wave—like’ morphologies compared to those with origins towards the surface of cortex.

## Conclusions

The propagation analyses presented here suggest that the small number of events of neocortical origin seen following hippocampectomy may not be of clinical significance owing to their highly localised nature and absence of overt spread to other brain regions. They also reinforce the suggestion that the perirhinal cortex may be an effective, putative target region for surgical intervention in temporal lobe epilepsy. In addition, these preliminary data suggest that it may be possible to identify whether seizure activity has a hippocampal focus by detailed analysis of neocortical activity alone.
